# Liver-Humanized NSG-PiZ Mice Support the Study of Chronic Hepatitis B Virus Infection and Antiviral Therapies

**DOI:** 10.1128/spectrum.05176-22

**Published:** 2023-05-18

**Authors:** Rossana Colón-Thillet, Daniel Stone, Michelle A. Loprieno, Lindsay Klouser, Pavitra Roychoudhury, Tracy K. Santo, Hong Xie, Laurence Stensland, Sarah L. Upham, Gregory Pepper, Meei-Li Huang, Martine Aubert, Keith R. Jerome

**Affiliations:** a Vaccine and Infectious Disease Division, Fred Hutchinson Cancer Center, Seattle, Washington, USA; b Department of Laboratory Medicine and Pathology, University of Washington, Seattle, Washington, USA; Cornell University College of Veterinary Medicine

**Keywords:** hepatitis B virus, engraftment, cccDNA, hepatocyte, adeno-associated virus, RTi

## Abstract

Hepatitis B virus (HBV) is a pathogen of major public health importance that is largely incurable once a chronic infection is established. Only humans and great apes are fully permissive to HBV infection, and this species restriction has impacted HBV research by limiting the utility of small animal models. To combat HBV species restrictions and enable more *in vivo* studies, liver-humanized mouse models have been developed that are permissive to HBV infection and replication. Unfortunately, these models can be difficult to establish and are expensive commercially, which has limited their academic use. As an alternative mouse model to study HBV, we evaluated liver-humanized NSG-PiZ mice and showed that they are fully permissive to HBV. HBV selectively replicates in human hepatocytes within chimeric livers, and HBV-positive (HBV^+^) mice secrete infectious virions and hepatitis B surface antigen (HBsAg) into blood while also harboring covalently closed circular DNA (cccDNA). HBV^+^ mice develop chronic infections lasting at least 169 days, which should enable the study of new curative therapies targeting chronic HBV, and respond to entecavir therapy. Furthermore, HBV^+^ human hepatocytes in NSG-PiZ mice can be transduced by AAV3b and AAV.LK03 vectors, which should enable the study of gene therapies that target HBV. In summary, our data demonstrate that liver-humanized NSG-PiZ mice can be used as a robust and cost-effective alternative to existing chronic hepatitis B (CHB) models and may enable more academic research labs to study HBV disease pathogenesis and antiviral therapy.

**IMPORTANCE** Liver-humanized mouse models have become the gold standard for the *in vivo* study of hepatitis B virus (HBV), yet their complexity and cost have prohibited widespread use of existing models in research. Here, we show that the NSG-PiZ liver-humanized mouse model, which is relatively inexpensive and simple to establish, can support chronic HBV infection. Infected mice are fully permissive to hepatitis B, supporting both active replication and spread, and can be used to study novel antiviral therapies. This model is a viable and cost-effective alternative to other liver-humanized mouse models that are used to study HBV.

## INTRODUCTION

Hepatitis B virus (HBV) remains a major cause of mortality and morbidity globally, even though global vaccine coverage with several highly effective vaccines is estimated to be 83% (1). The WHO estimates that only 10% of people infected with HBV are aware of their infection and that only 22% of these people are receiving antiviral therapies, such as reverse transcriptase inhibitors (RTi), that could potentially limit transmission (2). This has led to continued spread of the disease, particularly among children where vaccination rates are closer to 43%, and rates of mother-to-child transmission remain higher than desired (3). It is estimated that 1.5 million *de novo* HBV infections still occur annually, and since chronic hepatitis B (CHB) is a lifelong condition, it is likely that close to 300 million people currently have CHB (2–4). HBV contributes to approximately 820,000 deaths each year (2), as patients with advanced CHB are more likely to develop complications that may lead to death, such as cirrhosis or hepatocellular carcinoma (HCC). Notably, HBV is the second leading cancer-causing virus after human papillomavirus (HPV), and it is estimated that 3% of annual global cancer deaths are directly attributable to HBV (5). Thus, HBV remains a major public health concern, and a cure is desperately needed.

The study of HBV, both *in vitro* and *in vivo*, has been hampered by species restriction for HBV replication since only hepatocytes from humans, great apes, and Asian tree shrews (Tupaia belangeri) are fully susceptible to HBV infection (6). The differences in susceptibility across species are largely (although not entirely) driven by differences in the sodium taurocholate-cotransporting polypeptide (NTCP), the cellular receptor for HBV (7–13), which directly interacts with the Pre-S1 protein of HBV virions to mediate viral attachment and uptake. Since chimpanzee research has been phased out over the last 10 years, there are no animal models available to study HBV that fully replicate the situation in humans, where continuous virus replication and spread into uninfected hepatocytes occur in the presence of a functional host immune system. Nevertheless, a wide range of different avian, mouse, woodchuck, and nonhuman primate (NHP) hepadnavirus models have been developed that recapitulate different aspects of the HBV life cycle (reviewed in 6 and 14 to 17).

In recent years, immune-deficient liver-humanized mouse models have become widely used for study of actively replicating HBV and antiviral therapies that target replication and spread of HBV *in vivo*. Livers in these mice are fully permissive to HBV replication and can harbor chronic HBV infection for the lifetime of the mouse. These models use mice with genetic backgrounds that selectively kill mouse hepatocytes, including TK-NOG (18), uPA-RAG (19), uPA-SCID/PXB (20, 21), and FRG (22) mice, which create an environment that is receptive to primary human hepatocyte (PHH) engraftment following transplantation. PHH engraftment levels are sufficient to support high-level replication of both laboratory and clinical HBV isolates (19, 23–27), enabling their use in the study of antiviral therapies (25, 28–32).

Unfortunately, the current liver-humanized mouse models are technically difficult to establish, and this has limited how widely they are used for research, especially in academic labs. It is expensive to buy animals that have been humanized commercially, and establishment of a breeding colony in-house can be difficult. For example, TK-NOG and uPA transgenic mice are challenging to breed due to male infertility or neonatal fatality due to constitutive uPA expression, whereas FRG mice require a breeding license and extensive cycled treatment with 2-(2-nitro-4-trifluoro-methylbenzoyl)-1,3-cyclohexedione (NTBC) plus pretreatment with a uPA-expressing adenovirus vector to stimulate high-level engraftment (6, 15). Therefore, in continuation of our work developing antiviral gene-editing therapies targeting HBV (31, 33), we investigated the recently described liver-humanized NSG-PiZ mouse as an alternative model to study HBV disease pathogenesis and therapy. NSG-PiZ mice can be bred easily, need no excessive animal husbandry, and only require injection with a hepatotoxic agent to precondition the liver prior to intrasplenic PHH transplantation (34, 35).

Here, we show that humanized NSG-PiZ mice are fully permissive to HBV replication. HBV-positive (HBV^+^) NSG-PiZ mice can be followed longitudinally for at least 169 days and can be monitored for levels of human albumin (huAlb), HBsAg, and viral loads and for intrahepatic viral DNA levels at necropsy. HBV^+^ mice are responsive to traditional RTi therapy, and HBV^+^ PHHs can be transduced by hepatotropic adeno-associated virus (AAV) vector capsids, which is of particular importance for the study of novel gene therapies that target HBV (36, 37). Taken together, our data demonstrate that liver-humanized NSG-PiZ mice are a robust and comparatively inexpensive model that can be readily used to study the pathogenesis of CHB and antiviral therapies.

## RESULTS

### PHH engraftment in NSG-PiZ mice.

**FIG 1:**
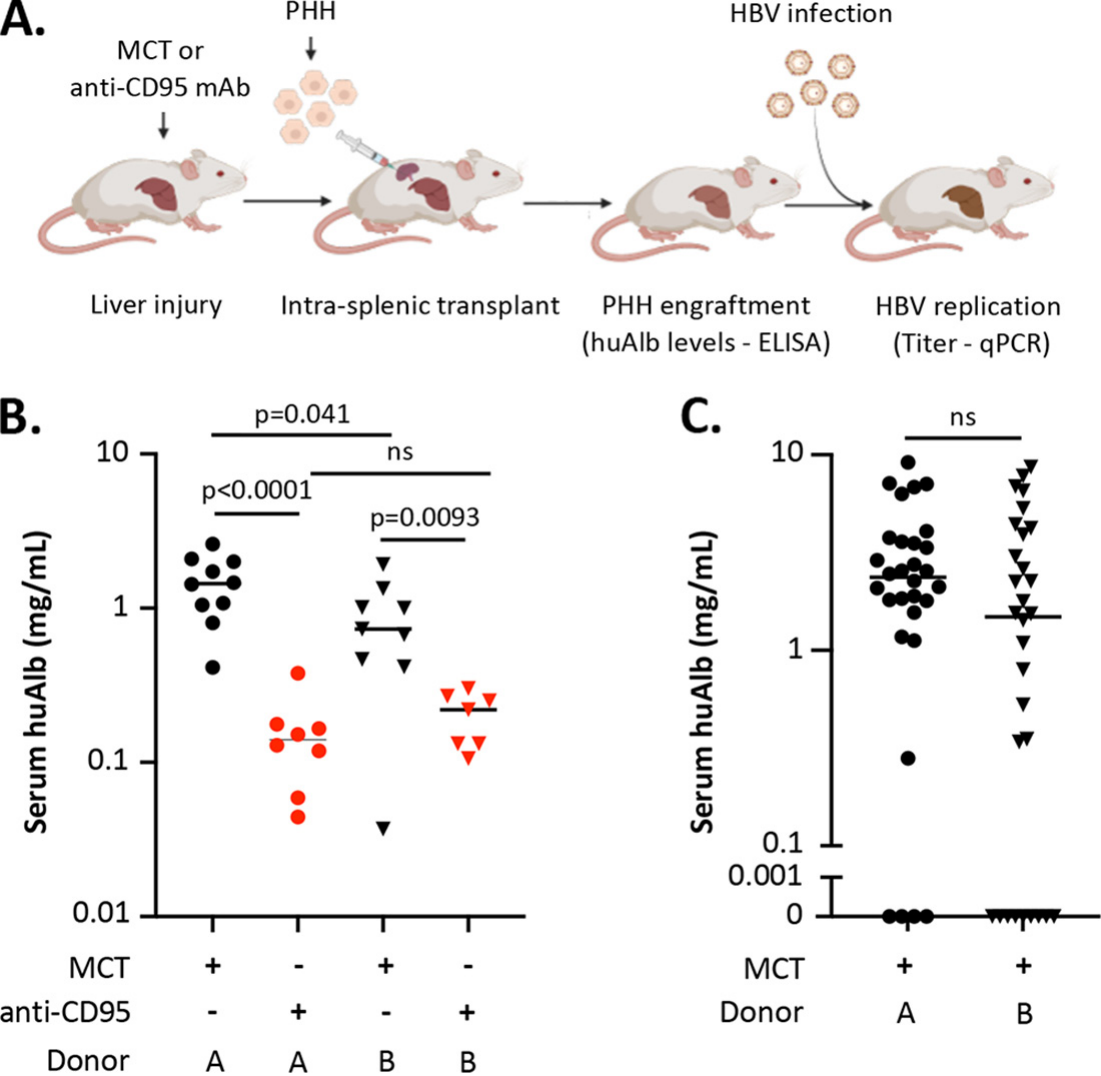
PHH engraftment in liver-humanized NSG-PiZ mice. (A) Experimental scheme for establishing HBV infection. (B) Male NSG-PiZ mice of 6 to 8 weeks of age were pretreated with either MCT (black) or anti-CD95 MAb (red) and then transplanted with 5 × 10^5^ PHHs from donor A (circles) or B (triangles). PHHs were administered to 10 mice per group (total, 40 mice), and 34/40 mice survived the transplant procedure and had detectable huAlb in serum. Serum levels of human albumin (huAlb) were determined at 12 weeks post-PHH transplant. (C) Male NSG-PiZ mice of 6 to 8 weeks of age were pretreated with MCT and then transplanted with 10^6^ PHHs from donor A (circles) or B (triangles). PHHs were administered to 30 mice per donor, and 47/60 mice (26, donor A; 21, donor B) survived the transplant procedure and had detectable huAlb in serum. Serum levels of huAlb were determined at 8 weeks post-PHH transplant. Student's *t* test was used to compare huAlb levels as indicated. Panel A was generated with biorender.com.

To evaluate the liver-humanized NSG-PiZ mouse model (35) for the study of HBV, we first evaluated the conditions required for optimal PHH engraftment (Fig. 1A). We pretreated 20 mice with an anti-CD95 monoclonal antibody (MAb) and 20 mice with the hepatotoxic plant-derived pyrrolizidine alkaloid monocrotaline (MCT) and then transplanted 10 mice from each group with 5 × 10^5^ PHHs from one of 2 pediatric donors (experiment 1; Table 1) and determined the efficiency of PHH engraftment. As a surrogate for liver repopulation by PHHs, we assessed human albumin (huAlb) at 12 weeks posttransplant (Fig. 1B). A total of 34/40 male mice survived the transplant procedure and had detectable huAlb in serum. MCT-pretreated mice had significantly higher huAlb levels than anti-CD95 MAb-pretreated mice (0.5 to 1 log, *P* < 0.0001 for donor A PHHs and *P* = 0.0093 for donor B PHHs), and donor A PHHs engrafted at slightly higher frequencies than donor B PHHs in MCT, but not anti-CD95 MAb-pretreated mice, although this difference was not significant when a single outlier was removed from the donor B PHH and MCT-treated group (*P* = 0.041 versus *P* = 0.083). We then assessed the efficiency of PHH engraftment in a larger cohort of 60 mice pretreated with MCT and transplanted with 1 × 10^6^ PHHs from donor A or B (experiment 2; Table 2). From this cohort, 47/60 mice (26, donor A; 21, donor B) survived the transplant procedure and had detectable serum huAlb at 8 weeks post-PHH transplant. Within this cohort, no significant difference in serum huAlb levels was seen between donors A and B (Fig. 1C).

**TABLE 1:** Details of experiment 1^*a*^

Group no.	Pretreatment	No. of mice per group	PHH donor and no. of PHHs per transplant	huAlb (mg/mL)^*b*^	HBV status and antiviral therapy
1	MCT	10	A, 5 × 10^5^	Fig. 1B	1, uninfected/untreated
					5, infected + ETV treatment
					4, infected + ETV+CPX treatment
2	Anti-CD95 MAb	10	A, 5 × 10^5^	Fig. 1B	1, uninfected/untreated
					4, infected + ETV treatment
					3, infected + ETV+CPX treatment
3	MCT	10	B, 5 × 10^5^	Fig. 1B	1, uninfected/untreated
					4, infected + ETV treatment
					5, infected + ETV+CPX treatment
4	Anti-CD95 MAb	10	B, 5 × 10^5^	Fig. 1B	1, uninfected/untreated
					3, infected + ETV treatment
					3, infected + ETV+CPX treatment

a In this experiment, a total of 34/40 male mice survived the transplant procedure and had detectable huAlb in serum. These are the mice from the experiments shown in Fig. 1B, Fig. 2A to G, Fig. 6A to H, Fig. 7A to D, and Fig. 8A to D. An experimental timeline for these mice is shown in Fig. 6A. huAlb levels were measured at 12 weeks post-PHH transplant.

b Data are shown in the cited figure.

**TABLE 2:** Details of experiment 2^*a*^

Group no.	Pretreatment	No. of mice per group	PHH donor and no. of PHHs per transplant	huAlb (mg/mL)^*b*^	HBV status and antiviral therapy
1	MCT	30	A, 1 × 10^6^	Fig. 1C	All uninfected
2	MCT	30	B, 1 × 10^6^	Fig. 1C	All uninfected

a In this experiment, a total of 47/60 male mice (26 donor A; 21 donor B) survived the transplant procedure and had detectable huAlb in serum. These are the mice from the experiment shown in Fig. 1C. huAlb levels were measured at 8 weeks post-PHH transplant.

b Data are shown in the cited figure.

### Histological analysis of chimeric mouse livers.

We analyzed the left lateral, right medial, and left medial lobes for hematoxylin and eosin (H&E)-stained livers from PHH-naive (PHH^−^) Swiss-Webster, PHH-naive (PHH^−^) NSG-PiZ, anti-CD95 MAb-treated PHH-naive (PHH^−^) NSG-PiZ, MCT treated PHH-naive (PHH^−^) NSG-PiZ, and MCT- or anti-CD95^−^ pretreated humanized (PHH^+^/HBV^−^) NSG-PiZ mice for gross pathology. Livers from PHH-naive NSG-PiZ mice and PHH-naive NSG-PiZ mice pretreated with anti-CD95 antibody or MCT showed abnormal hepatocyte pathology that was not seen in PHH-naive Swiss-Webster mice (Fig. 2A). There were also signs of inflammation and loss of endothelial integrity in PHH-naive NSG-PiZ and anti-CD95 antibody or MCT-preconditioned livers (data not shown). Humanized NSG-PiZ mouse livers (PHH^+^/HBV^−^) had a distinctive appearance in each lobe after H&E staining, similar to humanized uPA-SCID and TK-NOG mice (21, 25, 26, 38), which was independent of the pretreatment or PHH donor. We identified clusters of hepatocytes with a clear cytoplasm and a granular appearance that frequently contained large holes and were interspersed between darkly stained regions of hepatocytes with normal appearance (Fig. 2B). The clear hepatocytes appeared to have undergone microvesicular or macrovesicular steatosis and stained positive for lipid accumulation by Oil Red O (Fig. 2C).

**FIG 2:**
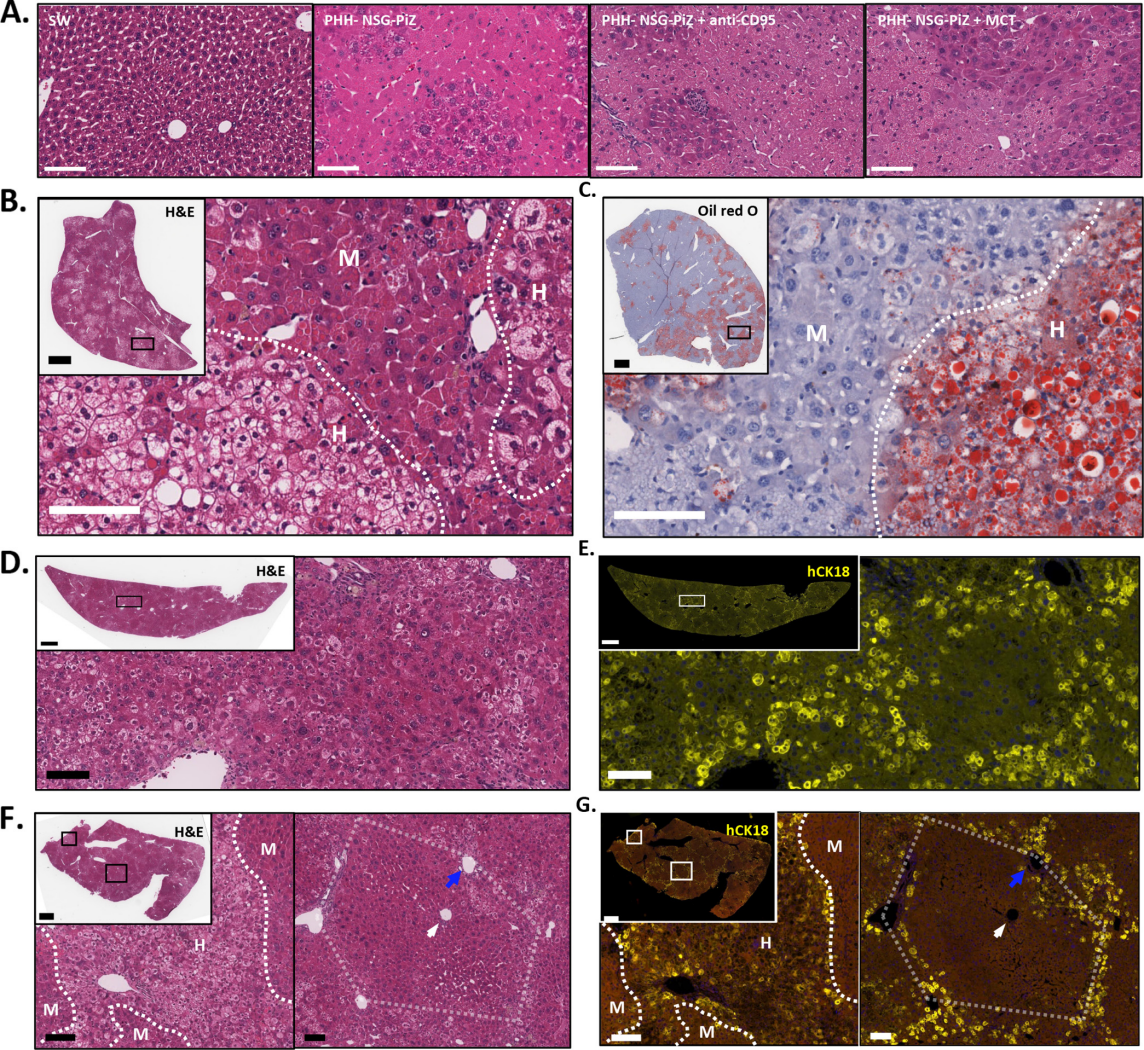
Control and humanized mouse liver phenotype. (A) H&E staining of control livers from a Swiss-Webster (SW) mouse, a PHH^−^ NSG-PiZ mouse, a PHH^−^ NSG-PiZ mouse euthanized 3 h after intravenous (i.v.) delivery of anti-CD95 MAb, and a PHH^−^ NSG-PiZ mouse euthanized after MCT treatment. Scale bars, 100 μm. (B and C) H&E (B)- or Oil Red O (C)-stained liver from an NSG-PiZ mouse pretreated with anti-CD95 MAb and then transplanted with 5 × 10^5^ PHHs from donor A; the mouse was euthanized at 139 days posttransplant. Approximate borders (dotted line) between human (H) and mouse (M) hepatocytes are indicated. Scale bars, 100 μm (main image) and 1 mm (inset image). (D and E) H&E staining (D) and hCK18 immunofluorescence (E) of serial liver sections from an NSG-PiZ mouse pretreated with MCT and then transplanted with 5 × 10^5^ PHHs from donor A; the mouse was euthanized at 233 days posttransplant. Scale bars, 100 μm (main image) and 1 mm (inset image). (F and G) H&E staining (F) and hCK18 immunofluorescence (G) in serial sections from the left lateral lobe of an NSG-PiZ mouse pretreated with MCT, transplanted with 5 × 10^5^ PHHs from donor A, and euthanized at 231 days posttransplant. Areas of liver containing human (H) or mouse (M) hepatocytes are shown. Human hepatocytes were predominantly periportal (blue arrows) or surrounding hepatic lobules (right panels), with fewer present within hepatic lobules or adjacent to the central vein (white arrowheads). Scale bars, 100 μm (left and right panels) and 1 mm (inset images).

We next performed immunofluorescence on chimeric livers to detect human cytokeratin 18 (hCK18) to determine which hepatocytes were of human origin and whether HBV replication is restricted to PHHs. The clear hepatocytes visible by H&E staining were mostly hCK18 positive (Fig. 2D and E) except for some within the center of densely packed clusters that looked morphologically identical but were weakly positive or negative for hCK18 (Fig. 2F and G, left images). In mice with lower or moderate levels of PHH engraftment, hCK18 staining was predominantly periportal or surrounding hepatic lobules containing mouse hepatocytes, and no hCK18-positive cells were detected around the central vein (Fig. 2F and G, right images).

### Kinetics of PHH engraftment and HBV infection in NSG-PiZ mice.

**FIG 3:**
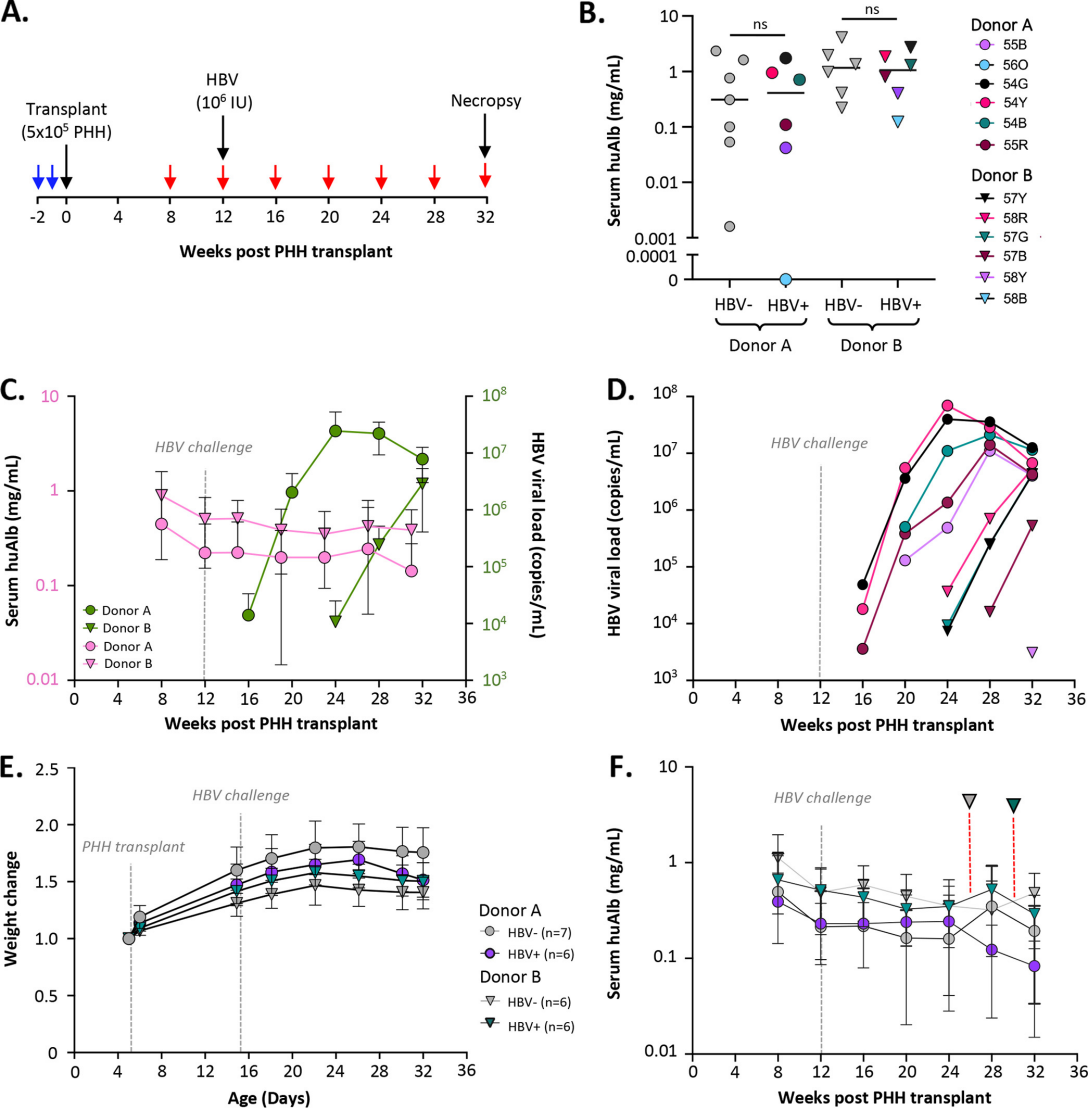
Longitudinal PHH engraftment and HBV replication in liver-humanized NSG-PiZ mice. (A) Experimental timeline showing MCT treatment (blue arrows) and blood draws (red arrows). (A to F) Male NSG-PiZ mice of 6 to 8 weeks of age were pretreated with MCT and then transplanted with 5 × 10^5^ PHHs (*n* = 13, donor A, circles; *n* = 12, donor B, triangles) and followed longitudinally. Half of the mice from each donor were HBV naive (gray symbols), and half were intravenously challenged with 10^6^ infectious units of a genotype D HBV clinical isolate at 12 weeks post-PHH transplant (colored symbols). All mice survived the transplant procedure, and 24/25 had detectable huAlb. (B) Experimental grouping (*n* = 6 to 7) based on huAlb levels at 8 weeks post-PHH transplant. HBV-negative mice, gray symbols; HBV-positive mice (colored symbols). HBV-challenged mice are color coordinated between panels B and D. (C) Mean serum huAlb levels (pink) and mean viral loads (green) for the HBV-challenged mice (*n* = 6) transplanted with PHHs from donor A (circles) or B (triangles) in panel B. (D) Longitudinal viral loads in serum over time for individual HBV^+^ mice in panel B. (E and F) Longitudinal mean weight change (E) and longitudinal mean serum huAlb levels (F) for the HBV-uninfected (gray) and HBV^+^ (colored) mice shown in panel B. Two mice transplanted with donor B PHH that died prematurely at days 183 and 215 posttransplant are indicated in panel F (dotted red lines). Student's *t* test was used to compare huAlb levels as indicated.

We investigated the long-term kinetics of PHH repopulation and HBV replication within the livers of HBV-infected and -naive NSG-PiZ mice (Fig. 3A). We transplanted 25 MCT-pretreated mice with PHH from donors A and B (experiment 3; Table 3) from which 24/25 mice survived the transplant procedure and had detectable serum huAlb at 8 weeks postengraftment. In the donor A group, 12 out of 13 mice had detectable levels of huAlb ranging from 0.002 to 2.34 mg/mL, whereas in the donor B group, 12 out of 12 mice had detectable huAlb levels ranging from 0.12 to 4.11 mg/mL (Fig. 3B).

**TABLE 3:** Details of experiment 3^*a*^

Group no.	Pretreatment	No. of mice per group	PHH donor and no. of PHHs per transplant	huAlb (mg/mL)^*b*^	HBV status and antiviral therapy
1	MCT	7	A, 5 × 10^5^	Fig. 3B, C, and F	7, uninfected
2	MCT	6	A, 5 × 10^5^	Fig. 3B, C, and F	6, infected with no treatment
3	MCT	6	B, 5 × 10^5^	Fig. 3B, C, and F	6, uninfected
4	MCT	6	B, 5 × 10^5^	Fig. 3B, C, and F	6, infected with no treatment

a In this experiment, a total of 24/25 male mice survived the transplant procedure and had detectable huAlb in serum. These are the mice from the experiments shown in Fig. 3A to F, Fig. 4A to C, and Fig. 5A to B. An experimental timeline for these mice is shown in Fig. 3A.

b Data are shown in the cited figure.

We asked whether PHH grafts remain stable in livers of HBV-infected and HBV-uninfected NSG-PiZ mice. We used huAlb levels to distribute mice into four experimental groups (A-uninfected, A-infected, B-uninfected, and B-infected) to normalize the levels of engraftment by donor (Fig. 3B) and intravenously challenged mice in groups A-infected and B-infected with a genotype D HBV clinical isolate at 12 weeks post-PHH transplant. Sera from all groups were collected every 4 weeks and analyzed for levels of huAlb and HBV DNA. HuAlb levels peaked at 8 weeks posttransplant in all groups as previously described for MCT-pretreated mice (35) and then slowly declined over time as expected (Fig. 3C and F) given previous work showing that Z alpha-1-antitrypsin levels in NSG-PiZ mice decrease as they age and the selective growth advantage this provides PHH is reduced (35). Mice receiving PHHs from donor B (infected and uninfected) had slightly higher levels of huAlb than mice receiving PHHs from donor A (~2-fold on average) at all time points. In groups A-infected and B-infected, 5 of 6 mice became viremic over the course of the experiment, and mice with the highest huAlb levels at week 8 post-PHH transplant had the highest viral loads (up to 7 × 10^7^ copies/mL) at approximately 12 weeks post-HBV challenge (Fig. 3C and D). HBV DNA was detected earlier in mice with higher huAlb levels independent of the PHH donor and was not detected at any time point in mice with huAlb levels below 0.04 mg/mL at 8 weeks post-PHH transplant. Unexpectedly, the onset of viremia was delayed by approximately 8 weeks for mice from group B-infected despite having higher huAlb levels than mice from group A-infected (Fig. 3C and F). No significant difference in huAlb levels was seen between infected and uninfected mice over time, and for both PHH donors, viral loads increased for at least 8 weeks. For the duration of the study, mouse weights remained stable in all treatment groups (Fig. 3E) and were slightly but not significantly higher in uninfected mice (Fig. 3E).

### Histological analysis of HBV^+^ chimeric mouse livers.

We performed immunofluorescence on HBV^+^ NSG-PiZ mouse livers from the same experiment (experiment 3; Table 3) to detect hCK18 and HBsAg to determine which hepatocytes were of human origin and whether HBV replication is restricted to PHHs (Fig. 4). Colabeling confirmed that HBV replication is restricted to hCK18-positive PHHs and was detected in all tested lobes within the liver (data not shown). Donor-dependent variation in the pattern of HBsAg staining was seen. In mice receiving PHHs from donor A, HBsAg was detected in most PHH within hCK18^+^ clusters (Fig. 4A), whereas in mice receiving PHHs from donor B, HBsAg was only detected in distinct foci within each hCK18^+^ cluster, predominantly in PHHs that were undergoing macrovesicular steatosis (Fig. 4B). This different pattern of HBsAg staining in donor B mice suggests viral spread through the cluster and could explain the delayed kinetics of viremia seen in these mice. Whether this spread of HBV into adjacent PHHs is NTCP dependent or independent is yet to be determined. Despite this apparent difference in HBV spread between HBV^+^ mice transplanted with PHHs from donors A and B, H&E-stained sections from HBV^+^ mice did not differ in their gross appearance (not shown). Livers from PHH^−^/HBV^−^, PHH^+^/HBV^−^, and PHH^+^/HBV^+^ NSG-PiZ mice were also stained with picrosirius red to visualize collagen and determine whether fibrosis occurs in humanized NSG-PiZ mouse livers (Fig. 4C). In PHH^−^/HBV^−^ mice, collagen staining was restricted to the borders of blood vessels. In contrast, collagen deposits in both PHH^+^/HBV^−^ and PHH^+^/HBV^+^ mice were also detected in highly vascularized areas of PHH engraftment.

**FIG 4:**
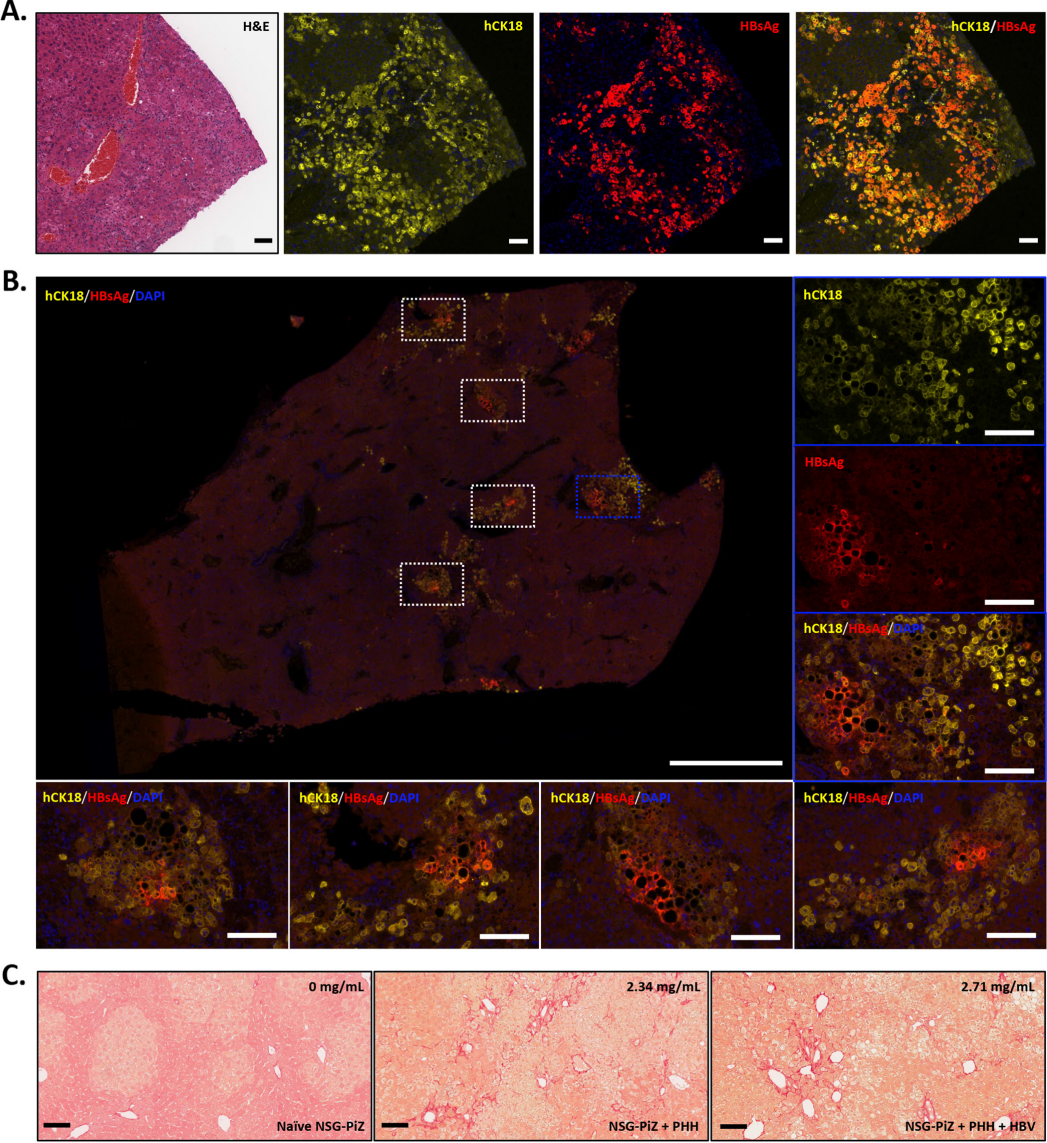
HBV replication and fibrosis in liver-humanized NSG-PiZ mice. (A) Liver from an NSG-PiZ mouse pretreated with MCT, transplanted with 5 × 10^5^ PHHs from donor A, and challenged intravenously with 10^6^ IU of a genotype D HBV clinical isolate. HBV challenge occurred at 10 weeks post-PHH transplant. Serial sections were stained with H&E (left panel) or via immunofluorescence with antibodies against hCK18 (yellow) and HBsAg (red). Scale bars, 100 μm. (B) Immunofluorescence in the liver of a male NSG-PiZ mouse pretreated with MCT, transplanted with 5 × 10^5^ PHHs from donor B, and challenged intravenously with 10^6^ IU of a genotype D HBV clinical isolate. HBV challenge occurred at 10 weeks post-PHH transplant. Merged (white boxes) or single-channel (blue box) images show DAPI (blue), HBsAg (red), and hCK18 (yellow) staining. Scale bars, 1 mm (large image) and 100 μm (smaller images). (C) Picrosirius red staining of livers from PHH^−^ NSG-PiZ mice or liver-humanized NSG-PiZ mice ± HBV. HBV challenge with 10^6^ IU of a genotype D HBV clinical isolate occurred at 12 weeks post-PHH transplant with 5 × 10^5^ PHHs from donor A, and humanized mice were euthanized at 242 days posttransplant (163 days post-HBV challenge). Serum huAlb levels at 8 weeks post-PHH are indicated. Scale bars, 100 μm.

**FIG 5:**
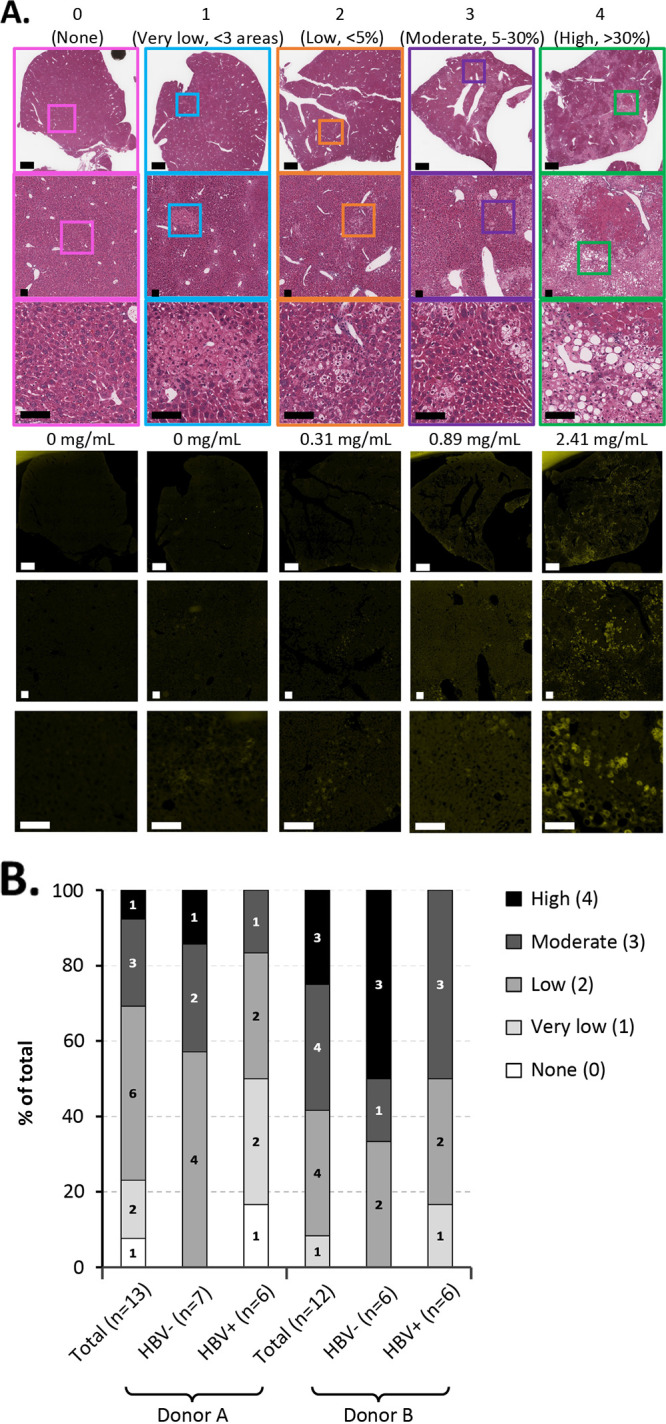
Long-term engraftment of PHH. (A) Scoring of PHH engraftment levels. Sections from the left lateral, left medial, and right medial liver lobes of each mouse were stained with H&E (top images) or for hCK18 expression by immunofluorescence (bottom images). Each mouse was scored from 0 to 4 for levels of PHH engraftment at necropsy as indicated. Scale bars, 1 mm (top image) and 100 μm (middle and bottom images). The respective huAlb levels at 8 weeks post-PHH transplant are shown for each example mouse. (B) PHH engraftment levels at necropsy or death for the 25 male mice transplanted with 5 × 10^5^ PHHs (±HBV infection) from the experiment described in Fig. 3. All mice were euthanized at day 242 post-PHH transplantation except for 2 mice that died at days 183 and 215 post-PHH transplant.

Although longitudinal huAlb levels indicate that peak PHH engraftment likely occurs from weeks 8 to 12 posttransplant, we determined the levels of engraftment at the study end for all 25 mice. We analyzed the levels of engraftment at necropsy to determine how the donor and HBV replication impacted longitudinal survival of PHH, even though peak engraftment of PHH likely occurred >20 weeks earlier when huAlb levels were ~0.5 log higher (Fig. 3C). Mice were euthanized at day 163 post-HBV infection (242 days posttransplant) except for two mice that died prematurely at days 106 (donor B, HBV negative) and 138 (donor B, HBV positive) post-HBV infection, respectively. We scored engraftment from 0 to 4 using serial sections from 3 lobes of each mouse liver stained with H&E and hCK18 as shown (Fig. 5A). Although our study was not powered sufficiently to determine significance, we saw a trend that mice receiving donor B PHHs had higher levels of engraftment than those receiving donor A PHHs (Fig. 5B). We also saw a trend that uninfected mice had higher levels of engraftment irrespective of the PHH donor, although further studies will be required to confirm this observation.

### Effects of single and combination drug antiviral therapy on HBV-positive NSG-PiZ mice.

**FIG 6:**
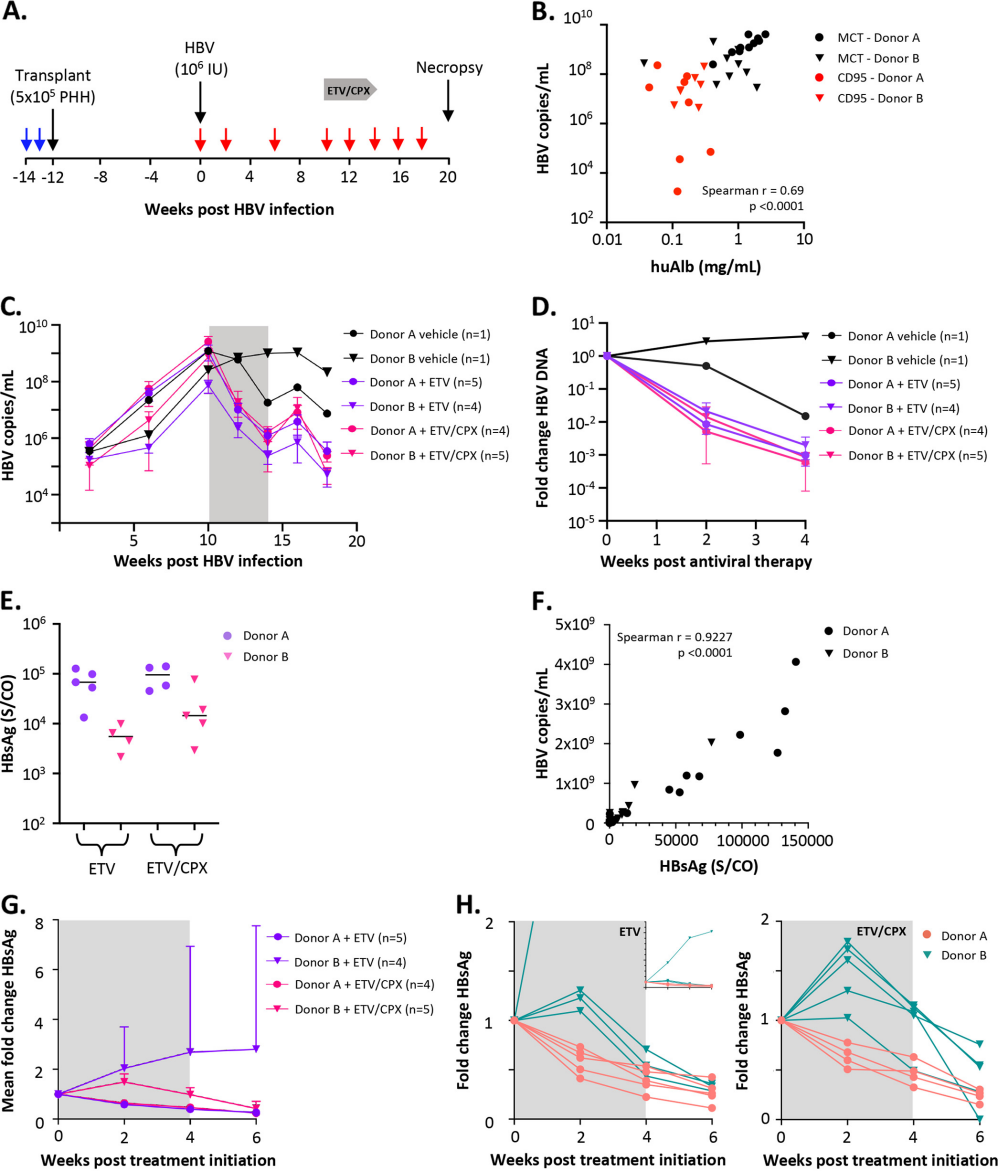
HBV-positive liver-humanized NSG-PiZ mice secrete HBsAg and respond to antiviral therapy. (A) Experimental timeline showing MCT or anti-CD95 MAb treatment (blue arrows), blood draws (red arrows), and antiviral entecavir (ETV) or entecavir-ciclopirox (ETV+CPX) treatment (gray horizontal arrow). (A to H) Antiviral therapy was assessed in the 6- to 8-week-old mice shown in Fig. 1B that were pretreated with MCT or anti-CD95 MAb and then transplanted with 5 × 10^5^ PHHs from donor A or B. Mice transplanted with PHHs from each donor were left uninfected (*n* = 1) or were infected with 10^6^ IU of a genotype D HBV clinical isolate at 12 weeks post-PHH transplant followed by 4 weeks of antiviral ETV or ETV+CPX drug therapy at 10 weeks post-HBV infection (*n* = 4 to 5). Mice were given ETV in drinking water (0.5 mg/kg) ± daily i.p. injections of CPX (5 mg/kg) as indicated for 4 weeks (gray bars). (B) Spearman’s correlation between the huAlb levels in serum at 12 weeks post-PHH transplant and the viral loads at 10 weeks post-HBV infection for the HBV-infected mice from Fig. 1B. (C) Longitudinal mean viral loads for MCT-pretreated, HBV-infected, and antiviral drug-treated mice. The gray box indicates a period of antiviral therapy. (D) Mean fold change in viral loads over 4 weeks of ETV or ETV+CPX antiviral therapy for MCT-pretreated, HBV-infected, and antiviral drug-treated mice. (E) Serum HBsAg levels at 10 weeks post-HBV infection for MCT-pretreated and HBV-infected mice. (F) Spearman’s correlation between the HBsAg levels in serum and the viral loads at 10 weeks post-HBV infection for MCT and anti-CD95 MAb-pretreated mice. (G) Mean fold change in serum HBsAg levels over 4 weeks of ETV or ETV+CPX antiviral therapy and 2 weeks of treatment withdrawal for MCT-pretreated, HBV-infected, and antiviral drug-treated mice. (H) Fold change in serum HBsAg levels for individual mice over 4 weeks of ETV or ETV+CPX antiviral therapy and 2 weeks of treatment withdrawal for MCT-pretreated, HBV-infected, and antiviral drug-treated mice. Error bars, SD.

We next evaluated the effects of the nucleoside RTi entecavir (ETV) alone or in combination with the capsid assembly inhibitor ciclopirox (CPX) in HBV-positive liver-humanized NSG-PiZ mice (Fig. 6A). CPX was included to determine whether antiviral synergy can occur when combined with ETV, as previously seen when combined with the RTi tenofovir in humanized uPA-SCID mice (39). For this study, we utilized the mice described for experiment 1 (Table 1; Fig. 1B) that were pretreated with MCT (Fig. 6/7) or anti-CD95 MAb (data not shown) transplanted with PHHs from donors A and B. Mice were inoculated intravenously with HBV at 10 weeks post-PHH engraftment, and HBV titers in serum were longitudinally monitored (Fig. 6C). At 10 weeks postinfection, HBV titers in MCT-pretreated mice peaked at approximately 10^8^ to 10^9^ copies/mL, and we found a strong positive correlation (Spearman’s *r* = 0.689) (Fig. 6B) between the peak HBV titers at week 10 postinfection and huAlb levels at week 12 posttransplant (2 weeks post-HBV infection) for all infected mice in this experiment (*n* = 34), suggesting that PHH engraftment rates are positively associated with HBV titers. When 3 outlier mice with low HBV titers were removed from this analysis, the correlation remained strong (*n* = 31, Spearman’s *r* = 0.676). At 10 weeks post-HBV infection, mice were administered ETV alone or in combination with CPX for 4 weeks to evaluate potential synergistic effects of HBV-targeted drug combinations on viral suppression. Treatment with ETV or ETV+CPX reduced viral titers by 1 to 3 log over 4 weeks for mice engrafted with PHHs from both donors, and no synergistic effect on viral suppression was seen (Fig. 6C and D). A reduction in viral titers was also seen in the donor A-transplanted vehicle control (*n* = 1), which confounds any conclusions for the 9 mice transplanted with donor A PHHs that received ETV or ETV+CPX. After antiviral therapy cessation, HBV titers rebounded ~1 log within 2 weeks but subsequently dropped another log at 4 weeks (Fig. 6C and D). A similar trend in viral load rebound kinetics was seen for the donor A-, but not donor B-, transplanted vehicle control mouse.

**FIG 7:**
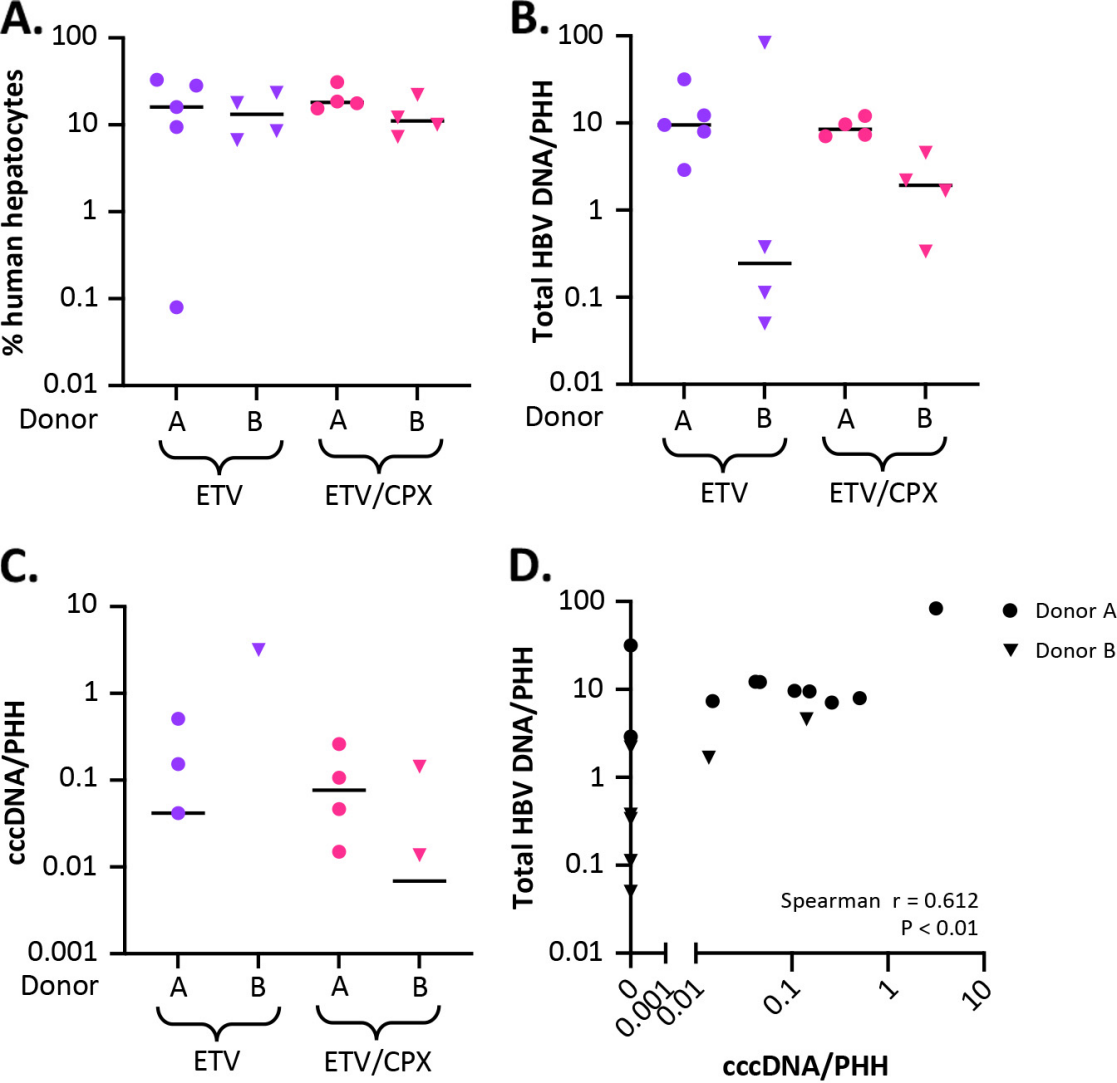
Human cell chimerism and liver-associated HBV DNA. (A) Levels of cell-associated human RPP30, mouse RPP30, total HBV DNA, and cccDNA in the liver at necropsy (20 weeks postinfection) were quantified by ddPCR (hRPP30, mRPP30, cccDNA) or qPCR (total HBV DNA) using DNA extracted via Qiagen kit or modified Hirt extraction (cccDNA) for the experiment described in Fig. 6. Levels of human hepatocyte chimerism (A), total HBV DNA (B), and cccDNA (C) were determined for MCT-pretreated mouse livers that received 4 weeks of ETV or ETV+CPX antiviral therapy. (D) Spearman’s correlation coefficients were calculated to determine whether levels of total cell-associated HBV DNA correlated with levels of cccDNA.

In parallel to viral load quantification, HBeAg, anti-HBeAg antibody, and HBsAg serum levels were measured in mice 10 weeks after HBV challenge and then followed longitudinally during the antiviral treatment phase and for 2 weeks after antiviral withdrawal. Neither HBeAg nor anti-HBeAg antibodies were detected in any of the mice in this study at any time point, so we analyzed the HBV genome for the presence of precore mutations by generating a consensus sequence from the challenge inoculum by next-generation sequencing (NGS) (GenBank accession no. OM194175). We found a previously described nonsense mutation in the precore region (TGG to TAG, G1898A/W28*) that prevents the synthesis of HBeAg and is found in 26% of 9,289 HBV genomes within the HBVdb database (40–45). For HBsAg, peak levels were seen at 10 weeks post-HBV infection and were higher in mice transplanted with donor A PHHs (Fig. 6E). A strong positive correlation was seen between serum HBsAg levels and viral loads (Fig. 6F). In all MCT-pretreated and donor A-transplanted mice, HBsAg levels progressively decreased at weeks 2, 4, and 6 posttreatment with ETV or ETV+CPX (Fig. 6G and H). Levels of HBsAg in 8 of 9 MCT-pretreated and donor B PHH-transplanted mice receiving ETV or ETV+CPX therapy increased slightly at 2 weeks posttherapy and then progressively decreased at weeks 4 and 6 posttherapy (Fig. 6H). No synergistic effect was seen in mice receiving CPX in addition to ETV irrespective of PHH donor. A single outlier mouse in the MCT+ETV experimental group complicated analysis of group trends regarding mean HBsAg fold change over time that was clear upon comparison of individual mice (Fig. 6G and H).

### Quantification of intrahepatic total HBV DNA and cccDNA.

At 20 weeks post-HBV infection, mice were euthanized, and levels of hRPP30, mRPP30, total HBV DNA, and covalently closed circular DNA (cccDNA) in liver quantified by droplet digital PCR (ddPCR) or quantitative PCR (qPCR) (total HBV DNA) for determination of hepatocyte chimerism and levels of PHH-associated total HBV DNA and cccDNA. At necropsy, levels of human hepatocyte chimerism were consistent for 32 of 33 mice (6.66 to 32.95%), with a single outlier showing ~2-log-lower chimerism (0.08%) (Fig. 7A), and all MCT-pretreated mice with detectable hRPP30 had detectable HBV DNA (0.05 to 84 copies/PHH) (Fig. 7B). Only 10 of 17 surviving MCT-pretreated mice had detectable cccDNA at necropsy (0.014 to 3.16 copies/PHH) (Fig. 7C), and all had total HBV DNA levels above 2.2 copies/PHH. The detection of cccDNA only in mice with higher total HBV DNA levels in liver may be related to the sensitivity of the modified Hirt extraction used in the cccDNA ddPCR. A newly described and widely validated protocol for cccDNA detection from the International Coalition to Eliminate HBV (46) may increase sensitivity for future work. Nevertheless, at the time of death, a positive correlation was seen between levels of total HBV DNA and levels of cccDNA in MCT-pretreated mice (Spearman’s *r* = 0.612) (Fig. 7D).

### AAV transduction of HBV^+^ PHHs in liver-humanized NSG-PiZ mice.

**FIG 8:**
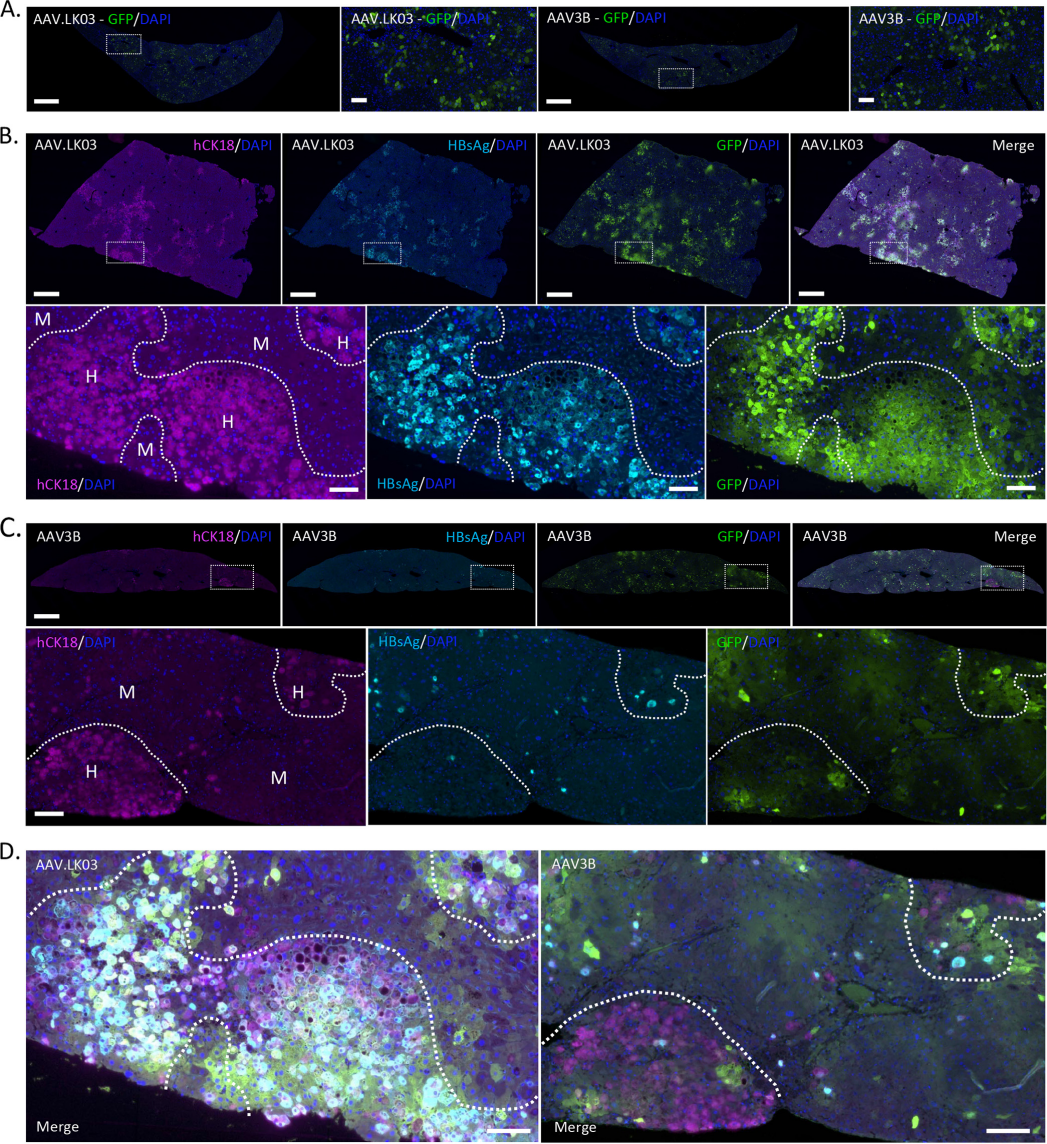
AAV transduction of HBV^+^ PHHs in liver-humanized NSG-PiZ mice. (A) Immunofluorescence imaging of GFP expression in control livers from untransplanted NSG-PiZ mice 28 days after intravenous administration of 10^12^ vg of scAAV.LK03-smCBA-GFP or scAAV3b-smCBA-GFP. Scale bars, 1 mm (low power) and 100 μm (high power). (B and C) Immunofluorescence labeling of GFP (green), hCK18 (purple), and HBsAg (cyan) in livers from NSG-PiZ mice pretreated with MCT, transplanted with 5 × 10^5^ PHHs from donor A (C) or B (B), challenged with 10^6^ IU of HBV, and then administered 10^12^ vg of scAAV.LK03-smCBA-GFP (B) or scAAV3b-smCBA-GFP (C). One mouse had huAlb levels of 1.01 mg/mL at 12 weeks post-PHH transplant and a viral load of 2.53 × 10^8^ IU/mL at 10 weeks post-HBV challenge (B), and the other mouse had huAlb levels of 0.8 mg/mL at 12 weeks post PHH transplant and a viral load of 7.74 × 10^8^ IU/mL at 10 weeks post-HBV challenge (C). For both mice, AAV was administered at 142 days post-HBV challenge (213 days post-PHH transplant), and euthanasia was performed at 27 days post-AAV administration (169 days post-HBV challenge, 240 days post-PHH transplant). Areas of human (H) and mouse (M) hepatocytes are delineated by a dotted line. Scale bars, 1 mm (low power) and 100 μm (high power). (D) Merged immunofluorescence images of the single channels shown at the bottom in panels B and C. Scale bars, 100 μm.

AAV vectors can deliver antiviral gene therapeutics to hepatocytes for the treatment of HBV (36, 37). To determine whether HBV^+^ PHHs in our model can be transduced by AAV vectors, we administered scAAV.LK03-smCBA-GFP or scAAV3B-smCBA-GFP, which have known tropism for PHHs in liver-humanized FRG and NSG-PiZ mice, respectively (47–49), intravenously at doses of 1 × 10^12^, 2 × 10^11^, and 2 × 10^10^ vector genomes (vg)/mouse to humanized NSG-PiZ mice that had been chronically infected with HBV for 142 days (experiment 4; Table 4). PHH-naive NSG-PiZ mice were only administered the highest dose for each AAV vector. Green fluorescent protein (GFP)-positive mouse hepatocytes were seen throughout the livers of PHH-naive NSG-PiZ mice 4 weeks after administration of scAAV.LK03-smCBA-GFP or scAAV3B-smCBA-GFP (Fig. 8A). In HBV^+^ mice receiving 10^12^ vg scAAV.LK03-smCBA-GFP per animal, GFP-positive mouse hepatocytes were also seen throughout the liver, but GFP expression was highly enriched in hCK18^+^/HBsAg^+^ PHHs (Fig. 8B). In contrast, scAAV3B-smCBA-GFP-treated PHH^+^/HBV^+^ NSG-PiZ mice had GFP-positive mouse hepatocytes throughout the liver at 1 × 10^12^ vg/mouse, but GFP expression was not enriched in PHHs, which contrasts with a previous report of enriched PHH transduction by an AAV3B vector in humanized NSG-PiZ mice (49). GFP^+^ hCK18^+^/HBsAg^+^ PHHs were detected, but at a much lower frequency than in mice receiving AAV.LK03-smCBA-GFP (Fig. 8C). For both AAV serotypes, fewer mouse hepatocytes and PHHs were transduced at doses of 2 × 10^11^ and 2 × 10^10^ vg/mouse (data not shown).

**TABLE 4:** Details of experiment 4^*a*^

Group no.	Pretreatment	No. of mice per group	PHH donor and no. of PHHs per transplant	huAlb (mg/mL)	HBV status	AAV vector and dose (vg/mouse)
1	None	1	None	0	None	scAAV3B-smCBA-GFP, 10^12^
2	None	1	None	0	None	scAAV.LK03-smCBA-GFP, 10^12^
3	MCT	3	A and B, 5 × 10^5^	>0.1^*b*^	3, infected	scAAV3B-smCBA-GFP, 2 × 10^10^
4	MCT	3	A and B, 5 × 10^5^	>0.1^*b*^	3, infected	scAAV3B-smCBA-GFP, 2 × 10^11^
5	MCT	3	A and B, 5 × 10^5^	>0.1^*b*^	3, infected	scAAV3B-smCBA-GFP, 10^12^
6	MCT	3	A and B, 5 × 10^5^	>0.1^*b*^	3, infected	scAAV.LK03-smCBA-GFP, 2 × 10^10^
7	MCT	3	A and B, 5 × 10^5^	>0.1^*b*^	3, infected	scAAV.LK03-smCBA-GFP, 2 × 10^11^
8	MCT	3	A and B, 5 × 10^5^	>0.1^*b*^	3, infected	scAAV.LK03-smCBA-GFP, 10^12^

a In this experiment, a single naive (nonhumanized) NSG-PiZ mouse was transduced with the high dose of each AAV vector (groups 1 and 2). A total of 18 male HBV^+^ mice were randomly assigned to groups 3 to 8 (at least one donor A PHH and one donor B PHH mouse per/group). At day 142 post-HBV infection, mice were administered AAV vectors intravenously at the indicated dose, and at 4 weeks post-AAV administration, livers were analyzed for levels of gene transfer (GFP expression).

b These huAlb values are from 12 weeks posttransplant. huAlb levels at the time of AAV administration were unknown.

## DISCUSSION

In the manuscript, we demonstrate that liver-humanized NSG-PiZ mice are fully permissive to infection by HBV, which selectively replicates in engrafted human hepatocytes and can establish chronic infections lasting at least 6 months. The infected livers contain cccDNA, secrete HBsAg, and produce infectious virus that has now been serially passaged through three generations of mice without loss of infectivity. We also show that viral loads in chronically infected mice are reduced following treatment with the RTi entecavir, a front-line therapy for patients with CHB, and that HBV-infected PHHs can be efficiently transduced by AAV vectors, which allows this model to be used in the study of novel small-molecule antivirals or gene-based curative therapies targeting HBV.

Liver-humanized mice have become the gold standard for the study of HBV replication and therapy, and several liver-humanized mouse models are available (6, 15). However, the complexity and/or cost of the most widely used uPA-SCID, FRG, and TK-NOG models have limited their use, and alternative models are still needed. We therefore investigated the NSG-PiZ mouse since it has several attributes that suggest it could be a robust and alternative model for the study of HBV. NSG-PiZ mice can be easily humanized via intrasplenic PHH injection (34, 35, 49); are readily available and affordable from commercial vendors; are viable and fertile; do not require a breeding license for academic use; are homozygous for the *SERPINA1* PiZ allele, ensuring all-transgenic offspring; do not die as neonates without PHH transplantation due to toxic transgene expression; and do not require adenovirus-uPA administration or drug conditioning posttransplant to promote high-level PHH engraftment. We now demonstrate that liver-humanized NSG-PiZ mice are fully permissive to HBV infection, which can persist for at least 6 months. Levels of HBV replication are comparable to other liver-humanized mouse models and strongly correlate with levels of PHH engraftment, and active infections can be established in mice with relatively low levels of PHH engraftment (huAlb > 0.1 mg/mL or <1% PHH engraftment). Thus, NSG-PiZ mice may be used to study disease progression and antiviral therapies during both acute and chronic HBV infections.

During our studies, we noticed that the kinetics of HBV replication was delayed in mice receiving PHHs from donor B relative to donor A, although the levels of peak viremia achieved were similar. Delayed replication occurred despite donor B mice having ~2-fold-higher huAlb levels, a similar decrease in huAlb levels over 30 weeks of monitoring, and equivalent levels of human DNA at necropsy to donor A mice. Multiple factors, including both host-intrinsic factors as well as factors related to hepatocyte processing, could account for such differences. Nevertheless, our observations suggest that donor B PHHs are, at least initially, less permissive to HBV infection, which is supported by our observations in HBV^+^ livers at necropsy. In donor A-transplanted mice, most hCK18^+^ PHHs were HBsAg^+^ with a diffuse staining pattern observed in areas of engrafted PHHs, whereas donor B PHH-transplanted mice contained HBsAg^+^ replication foci that appeared to be spreading through areas of uninfected hCK18^+^ PHHs. Most HBsAg^+^ PHHs within these foci were either undergoing macrovesicular steatosis or were adjacent to HBsAg^+^ PHHs that were undergoing macrovesicular steatosis, which presents a potentially novel mechanism of viral spread for HBV. It has previously been shown that polyploid and binuclear hepatocytes are more prevalent in patients with more severe CHB infections (50), and it is possible that HBV spread due to hepatocyte fusion is occurring in mice containing donor B PHHs that are undergoing macrovesicular steatosis. Whether the observed viral spread in mice receiving donor B PHHs is receptor dependent or independent is yet to be determined, and future studies will establish the role played by NTCP and other factors, including host restriction factors that limit HBV replication.

Overall, our results provide broad evidence that liver-humanized NSG-PiZ mice offer a robust alternative to existing models for the study of CHB and novel curative therapies. Furthermore, this model could enable broader access to HBV research in a more clinically relevant mouse model of disease.

## MATERIALS AND METHODS

### Cell culture.

Human embryonic kidney (HEK) 293 cells (51) were grown in Dulbecco’s modified Eagle’s medium (DMEM; Thermo Fisher, Waltham, MA) supplemented with 10% fetal calf serum.

### AAV vectors.

AAV vectors were generated by transiently transfecting HEK293 cells using polyethyleneimine (PEI) according to the method of Choi et al. (52). Briefly, HEK293 cells were transfected with AAV vector plasmid pscAAV-smCBA-GFP (53) in combination with the plasmid pRepCap3b, which contains Rep and Cap from AAV serotype 3b (kindly provided by David Russell [54]) or a plasmid that contains the AAV2 rep and AAV-LK03 capsid proteins, and a helper plasmid that expresses adenovirus helper proteins. At 24 h posttransfection, the medium was changed to serum-free DMEM, and after 72 h, cells were collected and resuspended in AAV lysis buffer (50 mM Tris, 150 mM NaCl, pH 8.5) before freeze-thawing 4 times. AAV stocks were purified by iodixanol gradient separation (52, 55) followed by ultrafiltration and concentration into phosphate-buffered saline (PBS) using an Amicon Ultra-15 column (EMD Millipore, Burlington, MA) before storage at −80°C. All AAV vector stocks were quantified by quantitative PCR using primers against the AAV inverted terminal repeat, with linearized plasmid DNA as a standard, according to the method of Aurnhammer et al. (56). AAV stocks were treated with DNase I (Thermo Fisher) and proteinase K (Thermo Fisher) prior to quantification.

### Animal welfare statement.

All animals were housed at the Fred Hutchinson Cancer Center, and all experimental procedures performed were reviewed and approved by the Institutional Animal Care and Use Committee (IACUC) of the Fred Hutchinson Cancer Center (protocol no. 51064). This study was carried out in accordance with the recommendations in the Guide for the Care and Use of Laboratory Animals of the National Institutes of Health (“The Guide”). All staff involved in animal work at Fred Hutch undergo appropriate training, as determined by the Fred Hutch IACUC and the Fred Hutch Department of Comparative Medicine. Standard housing, diet, bedding, enrichment, and light/dark cycles were implemented under animal biosafety level 2 (ABSL2) containment. Mice were monitored daily by animal technicians for overall well-being and basic husbandry parameters (for example, food intake, activity, signs of distress, stool consistency, and overall appearance), as well as daily observation by a veterinary technician and/or veterinarian. Retro-orbital blood draws or injections were performed after mice were anesthetized with isoflurane, and the presence or absence of deep pain was tested by the toe-pinch reflex, and the absence of response (leg flexion) to this test indicated adequate anesthesia. For intrasplenic transplant recovery surgeries, similar monitoring parameters were used, and anesthesia was tested by the loss of palpebral reflexes (eye blink). Slow-release analgesia (Buprenorphine SR) was administered prior to hepatocyte transplantation, and additional pain relief was provided throughout the study at the discretion of the clinical veterinarian based on clinical signs.

### Mice.

Male NSG-PiZ [NOD.Cg-*Prkdc^scid^* Il2rg*^tm^*^1Wjl^ Tg(SERPINA1*E342K)#Slcw/SzJ] mice were obtained from Jackson Laboratories (Bar Harbor, ME; strain no. 028842). Mice were housed in an ABSL2 facility and received standard housing, diet, bedding, enrichment, and light/dark cycles. Mouse weights were monitored for the duration of each experiment.

### Liver humanization.

Livers of NSG-PiZ mice were humanized according to the method of Borel et al. with modifications (35). Briefly, mice were pretreated to create an environment amenable to human hepatocyte engraftment via intraperitoneal (i.p.) injection with 100 μL of the hepatotoxin monocrotaline (MCT, 50 mg/kg) at 7 and 14 days prior to human hepatocyte transplant or via retro-orbital injection with 1 μg of an anti-CD95 antibody on the day of human hepatocyte transplant. Mice were administered Buprenorphine SR on the day prior to surgery to provide ~72 h of analgesia. On the day of transplant, 6- to 10-week-old mice were anesthetized with isoflurane, and then an incision was made in the left flank below the rib cage to expose the spleen. A total of 5 × 10^5^ or 1 × 10^6^ primary human hepatocytes were then slowly injected into the exposed spleen in a volume of 50 μL of sterile 1× Hanks balanced saline (Thermo Fisher) using a 27G 0.5-mL insulin syringe (Easy Touch, Houston, TX). The needle was left in the spleen for 30 s after injection before slow removal, and pressure was applied to the injection site with a sterile cotton swab to limit bleeding. The spleen was then returned to the abdominal cavity, which was closed with a 4-0 Vicryl suture (Ethicon, Cincinnati, OH), and the skin was closed with wound clips.

### Human hepatocytes.

Primary human hepatocytes were obtained from BioIVT (Westbury, NY) from a 10-month-old male African American donor (donor A) and a 3-year-old male Caucasian donor (donor B). The cause of death for both donors was a motor vehicle accident, and BioIVT provided cell viabilities, which, after thawing, for each donor were 83% and 96%, respectively. PHHs from both donors were reported to have metabolic activity for 14 liver enzymes *in vitro* and can reach >97% confluence within 5 days of culture. Cryo-preserved cells were thawed according to the supplier’s instructions before resuspension in sterile 1× Hanks balanced saline.

### HBV isolate.

HBV^+^ serum was obtained from BioIVT (Westbury, NY; human serum HMN13090). To determine the genotype and consensus sequence of the clinical isolate used in our study, we used a probe capture approach previously described for capture of HSV and HHV-6 (57, 58) to obtain a >10× coverage consensus HBV sequence. Briefly, tiled probes (XGen custom hybridization probe panel; IDT, Coralville, IA) were generated against the NCBI reference HBV genome (GenBank accession no. NC_003977) and used to capture HBV DNA, which was then sequenced via NGS. Significant heterogeneity was seen at multiple locations within the HMN13090 HBV genome, suggesting a diverse viral quasispecies was present within our “founder” clinical isolate. HMN13090 has been serially passaged through 3 generations of NSG-PiZ mice, using serum pooled from HBV^+^ mice to create a challenge inoculum for subsequent mice of which we determined the titer by qPCR as indicated below. For HBV challenge, HBV^+^ serum diluted in sterile 1× PBS was administered at a dose of 1× 10^6^ genome equivalents (copies) per animal via retro-orbital injection in a volume of 50 μL. The sequence for HBV isolate HMN13090 contains an in frame TGG to TAG (W to STOP) mutation G1898A in PreCore (40) that is highly prevalent in genome sequences from the HBVdb database (41). This mutation, also known as G1896A or W28*, stabilizes the HBV stem-loop (42), was previously implicated in the development of cirrhosis and HCC in advanced disease (43), and has been shown to emerge shortly before or around the time of HBe seroconversion (42, 44, 45).

### AAV gene transfer.

scAAV.LK03-smcBA-GFP or scAAV3B-smCBA-GFP vectors were delivered to naive (not humanized) NSG-PiZ mice at 10^12^ vector genomes per mouse (*n* = 1) or to HBV-positive liver-humanized NSG-PiZ mice at doses of 2× 10^10^, 2 ×10^11^, or 1 × 10^12^ vector genomes per mouse (*n* = 3/dose) via retro-orbital injection in a volume of 50 μL diluted in USP 1× PBS. Mice were euthanized 27 days later to analyze liver transduction.

### Drugs and antibodies.

MCT was obtained from Oakwood Chemicals (Estill, SC; item no. 002602), and 187.5 mg was dissolved in 1.8 mL of 1 M HCl followed by addition of 3 to 4 mL of distilled water. This solution was adjusted to pH 7.4 using 1 M NaOH solution and filled up to 15 mL with distilled water to make a stock solution of 12.5 mg/mL. Purified NA/LE hamster anti-mouse anti-CD95 antibody was obtained from BD Biosciences (clone Jo2 [RUO]; catalog no. 554254). Entecavir was obtained from Toronto Chemicals (Toronto, ON, Canada; catalog no. E558900) and was resuspended in MediDrop sucralose (clear H_2_O; product code 75-01-1001) and administered orally at 0.5 mg/kg/day. Ciclopirox was obtained from Bio-Techne/Tocris (Minneapolis, MN; catalog no. 6384) and was resuspended in 4% ethanol, 5.2% Tween 80, and 5.2% polyethylene glycol 400 (PEG 400) in PBS and administered daily at 5 mg/kg via i.p. injection.

### Human albumin quantification.

Human albumin levels in mouse sera were determined using the human albumin enzyme-linked immunosorbent assay (ELISA) kit (Bethyl Laboratories; catalog no. E88-129).

### HBsAg and HBeAg quantification.

HBsAg quantifications were performed on the Abbott Architect i2000 using the HBsAg qualitative (Abbott Laboratories, Des Plaines, IL; product no. B4P530) and HBsAg qualitative confirmatory (Abbott Laboratories, Des Plaines, IL; product no. B4P540) systems. Specimens with signal-to-cutoff (S/CO) ratios of >1.0 were deemed positive. The limit of detection (LOD) for quantification of HBsAg was 0.0524 IU/mL. Anti-HBeAg antibodies and HBeAg were quantified using the ETI-AB-EBK Plus (anti-HBe) kit (Diasorin, Cypress, CA; product no. P001929) or the ETI-EBK Plus (HBeAg) kit (Diasorin, Cypress, CA; product no. P001930), respectively. Specimens with signal-to-cutoff (S/CO) ratios of >1.0 were deemed positive.

### Quantitative PCR.

Total HBV DNA was detected in DNA extracted from serum using the MagNA Pure 96 system (Roche, Basel, Switzerland). Total HBV DNA, HBV cccDNA, human RPP30, and mouse RPP30 were all detected in DNA extracted from chimeric humanized NSG-PiZ mouse livers. For total HBV DNA, human RPP30 (hRPP30), and mouse RPP30 (mRPP30), droplet digital PCR (ddPCR) was performed using genomic DNA extracted using the DNeasy blood and tissue kit (Qiagen, Hilden, Germany). For cccDNA, ddPCR was performed using genomic DNA extracted via a modified Hirt procedure as described below, followed by treatment with T5 exonuclease (NEB, Ipswich, MA) at 37°C for 1 h. Primer/probe sets for HBV total DNA (31), human RPP30 (33), mouse RPP30 (31), and cccDNA (59) have been described previously. The LOD for quantification of HBV DNA by qPCR was 1.25 IU/reaction.

### Modified Hirt extraction.

The modified HIRT extraction was performed using a previously described protocol (60). Briefly, homogenized liver tissue extracts underwent a neutral-pH, 3-step alkaline lysis before the supernatant was passed over a Qiagen miniprep column to capture extrachromosomal DNA.

### Liver chimerism.

The percentages of human and mouse cells in chimeric livers were determined at death by quantifying human and mouse RPP30 levels by ddPCR as previously described (31), and the results were used to calculate the percentage of human hepatocytes using values previously reported for percentages of hepatocytes, Kupffer cells, sinusoidal endothelial cells, and stellate cells in livers of SCID mice and humanized uPA-SCID mice (38). The following assumptions were made: (i) nonhepatocyte mouse cells are found in regions of mouse and human liver tissue at different levels, (ii) levels of polyploid human and mouse hepatocytes were not different, (iii) all hRPP30 signals were exclusively from human hepatocytes, (iv) the levels of hepatocytes, Kupffer cells, sinusoidal endothelial cells, and stellate cells previously reported for SCID and uPA-SCID mice are for areas of liver that contain 100% mouse or 100% human hepatocytes (38), (v) in regions of human cells there are 0.58 nonhepatocyte mouse cells per human hepatocyte (38), and (vi) 34.2% of the mRPP30 signal that is not associated with human hepatocytes is derived from mouse hepatocytes (38). The following formula was used:

$${\text{HH}}_{\%}={\text{HH}}_{\text{total}}/\left({\text{HH}}_{\text{total}}+{\text{MH}}_{\text{total}}\right)\times 100$$

where HH_%_ is the percentage of all hepatocytes that are human in sample (level of human chimerism); HH_total_ is the total human hepatocyte number in sample, equal to the total hRPP30 copies in sample measured by ddPCR; MH_total_ is the total mouse hepatocyte number in sample, equal to mRPP30_Mo_ × 0.34; mRPP30_Hu_ is human tissue-associated mRPP30 signal (derived from nonhepatocyte mouse cells within regions of human graft), equal to HH_total_ × 0.58; mRPP30_Mo_ is mouse tissue-associated mRPP30 signal (derived from all nucleated mouse cell types in regions of mouse liver), equal to mRPP30_total_ − mRPP30_Hu_; and mRPP30_total_ is the total mRPP30 copies in sample measured by ddPCR.

### Histopathology and Oil Red O staining.

Mice were perfused with 1× PBS at necropsy, and tissues were either snap-frozen for DNA and RNA extraction, placed in 4% paraformaldehyde (PFA) overnight prior to embedding in paraffin, or postfixed in 4% PFA at 4°C overnight before then cryopreserving in 30% sucrose at 4°C overnight and embedding in OCT. Paraffin-embedded tissues were sectioned at 5 μm and then stained with hematoxylin and eosin. Frozen tissues were sectioned at 8 μm and stained with Oil Red O by the Fred Hutchinson Cancer Center, Experimental Histopathology Core. Oil Red O is a fat-soluble dye that stains neutral triglycerides and lipids in frozen sections and is routinely used to detect steatosis in liver sections (61).

### Immunofluorescence.

Immunofluorescence was performed on 5-μm paraffin sections. Sections were progressively hydrated in xylene, 100% ethanol, 95% ethanol, 70% ethanol, and 50% ethanol and then treated with citrate antigen retrieval buffer (10 mM sodium citrate, 0.05% Tween 20, pH 6.0) at 95°C for 30 min and blocked with 10% donkey serum with 1% bovine serum albumin (BSA) for 1 h. For staining of HBsAg, human cytokeratin 18 (hCK18), and GFP, sections were incubated with rabbit polyclonal anti-HBsAg (Bio-Rad, Hercules, CA; catalog no. OBT0990), mouse anti-hCK18 (Agilent, Santa Clara, CA; clone DC 10; catalog no. M701029-2), and goat polyclonal anti-GFP (Novus Biologicals, Centennial, CO; catalog no. NB100-1770). All primary antibody incubations were done in Tris-buffered saline (TBS) with 1% BSA at 4°C overnight. Sections were washed 2 times with TBS with 0.025% Triton X-100 and then incubated with secondary antibodies donkey anti-mouse Alexa Fluor 647 (Thermo Fisher; catalog no. A31571), donkey anti-rabbit Alexa Fluor 594 (Thermo Fisher; catalog no. A21207), and donkey anti-goat Alexa Fluor 488 (Abcam, Cambridge, UK; catalog no. ab150129) at room temperature for 1 h. Sections were counterstained with DAPI (4′,6-diamidino-2-phenylindole) before mounting with Prolong Gold antifade reagent (Thermo Fisher; catalog no. P36934).

### Microscopy.

Photomicrographs were obtained from the HALO Link platform (Indica Labs) using images acquired at 20× or 40× using the Leica Biosystems Aperio Versa 200 slide scanner.

### Data availability.

The sequence for HBV isolate HMN13090 has been uploaded to GenBank (accession no. OM194175).
